# VEGF-inhibitor switch trial in poor-responsive neovascular age-related macular degeneration: assessing brolucizumab vs. faricimab: VISTA study

**DOI:** 10.1007/s00417-025-07091-2

**Published:** 2026-01-12

**Authors:** Adnan Kilani, Denise Vogt, Vasiliki Moysidi, Abdelrahman Assaf, Armin Wolf, Efstathios Vounotrypidis

**Affiliations:** Department of Ophthalmology, Ulm University Hospital, Ulm, Prittwitzstraße 43, 89075 Germany

**Keywords:** Neovascular age-related macular degeneration, Anti-VEGF switch, Brolucizumab, Faricimab, Treat-and-extend, Real-world

## Abstract

**Purpose:**

To compare short-term (12 weeks) and long-term (48 weeks) anatomical and functional outcomes after switching to brolucizumab (BRZ) or faricimab (FAR) under a treat-and-extend (TAE) regimen without an initial loading phase in eyes with poor-responsive neovascular age-related macular degeneration (nAMD).

**Methods:**

This retrospective, single-center study included eyes with poor-responsive nAMD previously treated with anti-VEGF agents that were switched to BRZ or FAR between 2022 and 2025. Functional outcomes (best-corrected visual acuity [BCVA]) and anatomical parameters (central subfield thickness [CST], fibrovascular pigment epithelial detachment [fvPED] height, intraretinal/subretinal fluid [IRF/SRF], subretinal hyperreflective material [SHRM]) were assessed at baseline, week 12, and week 48. Injection frequency, interval extension, and safety were recorded.

**Results:**

Sixty-six eyes (41 BRZ, 25 FAR) were included. At 12 weeks, BCVA remained stable (BRZ 0.49 logMAR, FAR 0.40; *p* = 0.10). CST decreased significantly in both groups, greater with BRZ (243 μm vs. 280 μm; *p* = 0.04). Mean injections (2.95 vs. 3.2; *p* = 0.12) and intervals (5.9 vs. 5.6 weeks; *p* = 0.90) were comparable; no intraocular inflammation (IOI) occurred. At 48 weeks, BCVA was numerically better with FAR (0.20 vs. 0.49 logMAR; p = 0.08). CST reduction was sustained andcomparable (259 µm vs. 260 µm; p = 0.50). FAR achieved greater fvPED reduction (110 µm vs. 160 µm; p = 0.022). BRZ required fewer injections (6.3 vs. 7.2; p = 0.009) with similar intervals (9.4 vs. 9.9 weeks; p = 0.80). Mild IOI occurred in two BRZ eyes (4.9%) and none with FAR.

**Conclusions:**

In poor-responsive nAMD, switching to BRZ or FAR under a TAE regimen without upload achieved stable vision, sustained CST reduction, and comparable treatment burden over one year. FAR showed greater fvPED regression and a slight functional trend, while BRZ achieved faster fluid resolution with fewer injections.

## Abstract (fr)

**Key messages:**

***What is known*:**

- Recent clinical trials have shown that switching to newer anti-VEGF agents such as brolucizumab (BRZ) or faricimab (FAR) can improve anatomical outcomes and reduce treatment burden in poor-responsive neovascular age-related macular degeneration (nAMD).
- Most available real-world studies have evaluated only short-term outcomes, or long-term cohorts that included a treat-and-extend (TAE) regimen with an initial loading phase.
- To date, only one short-term head-to-head comparison between BRZ and FAR under real-world settings has been published.

***What is new*:**

- Our study revealed the first long-term real-world head-to-head comparison of BRZ and FAR in poor-responsive nAMD under a TAE regimen without an initial loading phase.
- Short-term (week 12 after switch) results showed significant fluid reduction and stable BCVA in both groups, with faster CST improvement under BRZ.
- Long-term (week 48 after switch) outcomes demonstrated sustained anatomical stability and comparable vision in both agents, with fewer injections under BRZ and greater fibrovascular pigment epithelial detachment (fvPED) reduction under FAR. 
- These results support a practical, resource-efficient treatment approach that maintains visual stability while reducing patient and clinic burden.

## Introduction

Neovascular age-related macular degeneration (nAMD) is a leading cause of irreversible vision loss in the elderly worldwide [1]. Intravitreal anti-vascular endothelial growth factor (anti-VEGF) therapy (IVT) has transformed its management, markedly reducing rates of severe visual impairment [2]. Nevertheless, most patients require long-term, frequent injections, and real-world outcomes often lag behind clinical trial results due to undertreatment or suboptimal response to IVT [2]. Up to 30–50% of treated eyes show persistent fluid despite regular therapy, classifying them as “poor responders” or “treatment-resistant” [3–5].

Switching to newer anti-VEGF agents in such cases is a widely adopted real-world strategy aimed at improving anatomical outcomes, maintaining or enhancing vision, and extending treatment intervals [5–13]. Two next-generation agents - brolucizumab (BRZ) and faricimab (FAR) - offer distinct molecular characteristics and potential advantages in this setting. Both have a high VEGF-A binding capacity and are administered at similar concentrations in the injection solution (120 mg/ml), resulting in comparable theoretical VEGF-A binding potential per injection despite differences in molecular size, structure, and pharmacokinetics [14, 15].

FAR is the first bispecific antibody targeting both VEGF-A and angiopoietin-2 (Ang-2), whereas BAR targets VEGF-A alone. While Ang-2 is a well-established pathogenic driver in diabetic macular edema (DME) and retinal vein occlusion (RVO), current evidence suggests that intraocular Ang-2 levels in nAMD are comparatively low and often not significantly elevated compared to controls [16–18]. This provides a unique opportunity to compare two potent anti-VEGF-dominant agents without a major confounding effect from Ang-2 blockade.

Although both agents have demonstrated robust anatomical improvements and the potential for extended dosing intervals in clinical trials and real-world practice [3, 5, 6, 8–15, 19], direct comparative long-term data in poor-responsive nAMD are scarce and to date there are no long-term data omitting the initial upload at switch [20]. To our knowledge, this is the first real-world long-term comparison of these agents in TAE without a loading phase to support personalized switching strategies. The **V**EGF-**I**nhibitor **S**witch **T**rial in Poor-Responsive nAMD: **A**ssessing BRZ vs. FAR (VISTA study) was designed to address this gap by retrospectively comparing short-term (12 weeks) and long-term (48 weeks) anatomical and functional outcomes under a treat-and-extend (TAE) regimen without an initial loading phase in eyes with documented poor response to prior anti-VEGF therapy.

## Materials and methods

### Study design and setting

This retrospective, single-centre, real-world cohort study was conducted and initiated at the Department of Ophthalmology, Ulm University Hospital (Germany) on 20 April 2020. The study adhered to the tenets of the Declaration of Helsinki and received ethical approval (application 468/23; decision February 14, 2024).

Short-term (12 weeks) and long-term (48 weeks) outcomes after switching IVT to BRZ or FAR (2022–2025) were directly compared in eyes with poor-responsive nAMD using a TAE regimen, without initial loading doses.

## Eligibility criteria

Eyes with poor-responsive nAMD to prior anti-VEGF agents were included. Poor-response eyes were defined as those with persistent intraretinal (IRF) or subretinal (SRF) fluid despite receiving at least three anti-VEGF injections at treatment intervals of no longer than six weeks. Exclusion criteria included diagnosis of ocular disease other than nAMD that may contribute to ME, iris or retinal neovascularization at baseline, baseline best-corrected visual acuity (BCVA) worse than 1.3 logMAR, significant media opacities (i.e., cataract, vitreous hemorrhage), uncontrolled glaucoma, other vitreoretinal pathology (i.e., vitreomacular traction, epiretinal membrane or macular hole on spectral-domain-optical coherence tomography [SD-OCT]), previous vitreoretinal surgery, a steroid intravitreal injection or implant at any time point.

## Treatment protocol

At the date of switch to either BRZ or FAR, prior to the 1st injection, a baseline visit was conducted to assess demographics (age, sex), the number of previous IVTs, and the last anti-VEGF agent injected with the last dosing interval (Table 1). Patients received either BRZ 6 mg/0.05 mL or FAR 6 mg/0.05 mL as intravitreal injection and were then managed under a TAE regimen without an initial loading phase. Injection intervals were extended by 2 weeks if there was no IRF or retinal hemorrhage and SRF was absent or reduced; intervals were reduced by 2 weeks if disease activity recurred, with a minimum interval of 4 weeks. A TAE regimen was applied without an initial upload to reduce treatment burden and to offer an individualized treatment. This protocol differs from the pivotal phase 3 trials (HAWK and HARRIER for BRZ; TENAYA and LUCERNE for FAR), which employed three to four consecutive monthly injections as a loading phase before interval extension [14, 15].

**Table 1:** Demographics and baseline ocular characteristics at treatment switch

Characteristics	BRZ Group (*n* = 41)	FAR Group (*n* = 25)
Age, mean (SD), years	78.6 (± 7.9), range: 62–93	79.6 (± 5.0), range: 68–87
Male : Female (n)	17 : 24	13 : 12
BCVA, median (IQR), LogMAR	0.6 (1.0-0.4.0.4)	0.4 (0.8 − 0.13)
CST, median (IQR), µm	313.0 (267.0–391.0.0.0)	315.0 (267.0–409.0.0.0)
OCT biomarkers present, % IRF SRF SHRM	66 80 56	72 64 60
fvPED maximum height, median (IQR), µm	210.0 (165.0–326.5.0.5)	151.0 (107.5–195.0)
Last anti-VEGF agent prior switch, % AFT : BRZ : BZB : FAR : RZB	49 : 0 : 19 : 0 : 32	48 : 8 : 8 : 0 : 36
Mean number of prior anti-VEGF injections (SD)	22.2 (± 13.6), range: 4–50	25.8 (± 21.6), range: 3–68
Mean last treatment interval prior switch (SD), weeks	5.2 (± 0.9), range: 4–6	4.9 (± 0.8), range: 4–6

Abbreviations: *AFT*: Aflibercept 2 mg; *BCVA*: best-corrected visual acuity; *BRZ*: Brolucizumab; *BZB*: Bevacizumab; *CST*: central subfield thickness; *FAR*: Faricimab; *fvPED*: fibrovascular pigment epithelial detachment;* IQR*: interquartile range; *RZB*: Ranibizumab; *SD*: standard deviation; *SHRM*: subretinal hyperreflective material

## Outcome measures

At the date of switch (baseline) and subsequent visits, patients underwent a comprehensive ophthalmological assessment including best corrected visual acuity (BCVA), spectral domain optical coherence tomography (SD-OCT), intraocular pressure (IOP) measurement, slit-lamp biomicroscopy and funduscopy following pupil dilatation prior to every injection after switch. BCVA was measured in Snellen decimal visual acuity and converted to logMAR for statistical analysis. SD-OCT acquisition was performed with the same device from baseline (either Spectralis HRA2 + OCT, Heidelberg Engineering or ZEISS CIRRUS 5000, Carl Zeiss AG). A comprehensive evaluation of central subfield thickness (CST) changes, maximum height of fibrovascular pigment epithelial detachment (fvPED), and the presence of OCT biomarkers such as IRF, SRF, and subretinal hyperreflective material (SHRM) was assessed by three retina specialists (A.K., D.V., and E.V.) and evaluated over a follow-up period of 48 weeks.

### Statistical analysis

Statistical analyses were performed using SPSS Statistics version 29.0.1.0 (IBM Corp., Armonk, NY, USA). Data distribution was assessed for normality prior to analysis. Depending on the distribution pattern, parametric or non-parametric tests were applied to evaluate changes in BCVA and OCT parameters between baseline, week 12, and week 48. Categorical variables were analyzed using the Chi-square test or Fisher’s exact test, as appropriate. Continuous variables are presented as mean ± standard deviation (SD) for normally distributed data or median with interquartile range (IQR) for non-normally distributed data. All statistical tests were two-tailed, and a p-value < 0.05 was considered statistically significant. A post-hoc power analysis, based on an assumed effect size of 0.5, an alpha level of 0.05, and a sample size of 27, indicated an achieved power of 0.81 for detecting within-group changes. With only one eye per patient enrolled, inter-eye correlation was not an issue in this dataset.

## Results

### Demographic and baseline characteristics

A total of 66 eyes from 66 patients with poor-responsive nAMD were included, of which 41 eyes were switched to BRZ and 25 eyes to FAR.

Baseline demographic and clinical characteristics were comparable between both groups (Table 1). The mean age was 78.6 ± 7.9 years (range: 62–93) in the BRZ group and 79.6 ± 5.0 years (range: 68–87) in the FAR group; *p* = 0.72. Baseline median BCVA was slightly higher in the FAR group 0.40 logMAR (IQR: 0.8 − 0.13) compared with BRZ 0.60 logMAR (IQR: 1.0-0.4.0.4); *p* = 0.31. The median CST was similar 313.0 μm (IQR: 267.0–391.0.0.0) vs. 315.0 μm (267.0–409.0.0.0); *p* = 0.67. Maximum fvPED height and the presence of IRF or SRF did not differ significantly between groups (*p* > 0.05).

All eyes had undergone extensive prior anti-VEGF therapy (mean ≈ 22–26 injections, most commonly aflibercept) and exhibited persistent exudation, confirming a poor-responsive, real-world cohort.

## Short-term outcomes (week 12 after switch)

After 12 weeks, both treatment groups demonstrated a slight improvement in visual acuity and a marked reduction in retinal fluid (Table 2). Median BCVA improved to 0.49 logMAR (IQR: 1.0-0.3.0.3) in the BRZ group and remained stable with 0.40 logMAR (IQR: 0.8 − 0.1) in the FAR group, without a statistically significant difference between groups (*p* = 0.10).

**Table 2:** Intergroup Comparison of Short-Term Outcomes (Week 12 After Switch)

Characteristics	**BRZ Group** (*n* = 41)	**FAR Group** (*n* = 25)	P-value
BCVA, median (IQR), LogMAR	0.49 (1.0-0.3.0.3)	0.4 (0.8 − 0.1)	0.1
CST, median (IQR), µm	243 (212–287)	280 (246–319)	0.04*
Change in CST, median (%)	−16.9	−9.7	0.1
OCT biomarkers present (%) IRF SRF SHRM	43.9 41.5 48.8	52 36 64	0.5 0.8 0.9
fvPED maximum height, median (IQR), µm	155.5 (123–230)	135 (70–185)	0.08
Change in fvPED maximum height, median (%)	−18.5	−5.2	**0.01**
Mean number of anti-VEGF injections (SD)	2.95 (± 0.6)	3.2 (± 0.7)	0.12
Mean last achieved treatment interval (SD), weeks	5.9 (± 2.4)	5.63 (± 1.5)	0.9
Mean interval extension, weeks	+ 0.7	+ 0.6	0.2
Adverse events consistent with IOI, n (%)	0	0	n.a.

Abbreviations: *AFT*: Aflibercept 2 mg; *BCVA*: best-corrected visual acuity; *BRZ*: Brolucizumab; *BZB*: Bevacizumab; *CST*: central subfield thickness; *FAR*: Faricimab; *fvPED*: fibrovascular pigment epithelial detachment; *IOI*: intraocular inflammation; *IQR*: interquartile range; *RZB*: Ranibizumab; *SD*: standard deviation; SHRM: subretinal hyperreflective material

* P-value for intergroup comparison: statistically significant

A significant anatomical improvement was observed in both groups. The reduction in median CST was significantly greater in the BRZ group (243 μm [IQR: 212–287] vs. 280 μm [IQR: 246–319]; *p* = 0.04), indicating a more pronounced short-term fluid resolution compared with FAR (Table 2). In contrast, FAR tended to induce greater fvPED regression (135 μm [70–185] vs. 155.5 μm [123–230]; *p* = 0.08), suggesting enhanced remodeling of the PED complex (Table 2). Although FAR showed lower absolute fvPED height at week 12 (135 μm [70–185] vs. 155.5 μm [123–230]; *p* = 0.08), the percentage-based analysis confirmed a significantly stronger early fvPED reduction in the BRZ group, along with a numerically greater CST decrease (Table 2). OCT biomarker evaluation showed comparable proportions of IRF/SRF between both treatments (*p* > 0.05).

The mean number of injections (2.95 ± 0.6 vs. 3.2 ± 0.7; *p* = 0.12) and most recent treatment interval (5.9 ± 2.4 vs. 5.6 ± 1.5 weeks; *p* = 0.90) were comparable (Table 2). No IOI was observed in either group.

### Long-term outcomes (week 48 after switch)

At 48 weeks, 88% of the 66 eyes completed the follow-up period. Six BRZ and two FAR eyes discontinued (10%) due to adverse events or loss to follow-up. (Table 3).

**Fig. 1:**
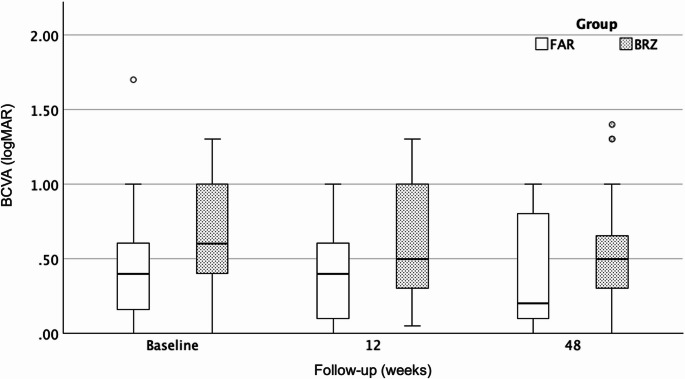
Boxplots showing best-corrected visual acuity (BCVA, logMAR) at baseline, week 12, and week 48 after switch to brolucizumab (BRZ) or faricimab (FAR). Data represent median ± interquartile range (IQR). BCVA remained stable in both groups, with a trend toward better visual acuity in the FAR group at week 48

Median BCVA remained stable with 0.49 logMAR in the BRZ group and improved to 0.20 logMAR in the FAR group, showing a trend toward better visual outcomes with FAR (*p* = 0.08) (Fig. 1; Table 3).

**Table 3:** Intergroup Comparison of Long-Term Outcomes (Week 48 After Switch)

Characteristics	**BRZ Group** (*n* = 35)	**FAR Group** (*n* = 23)	P-value
BCVA, median (IQR), LogMAR	0.49 (0.6 − 0.3)	0.2 (1.0-0.09.0.09)	0.08
CST, median (IQR), µm	259 (220–290)	260 (245–298)	0.5
Change in CST, median (%)	−14.3	−8.8	0.4
OCT biomarkers present (%) IRF SRF SHRM	41.5 41.5 34.1	40.0 24.0 40.0	0.7 0.08 0.7
fvPED maximum height, median (IQR), µm	160 (117–250)	110 (74–161)	0.022*
Change in fvPED maximum height, median (%)	−12.9	−15.7	0.2
Mean number of anti-VEGF injections (SD)	6.3 (± 0.83)	7.22 (± 1.5)	0.009*
Mean last achieved treatment interval (SD), weeks	9.37 (± 1.7)	9.9 (± 3.5)	0.8
Mean interval extension, weeks	+ 4.2	+ 5.0	0.3
Adverse events consistent with IOI, n (%)	2 (4.9)	0	n.a.

Abbreviations: *AFT*: Aflibercept 2 mg; *BCVA*: best-corrected visual acuity; *BRZ*: Brolucizumab; *BZB*: Bevacizumab; *CST*: central subfield thickness;* FAR*: Faricimab; *fvPED*: fibrovascular pigment epithelial detachment; *IOI*: intraocular inflammation; *IQR*: interquartile range, *ZB*: Ranibizumab;* SD*: standard deviation; *SHRM*: subretinal hyperreflective material

* P-value for intergroup comparison: statistically significant

**Fig. 2:**
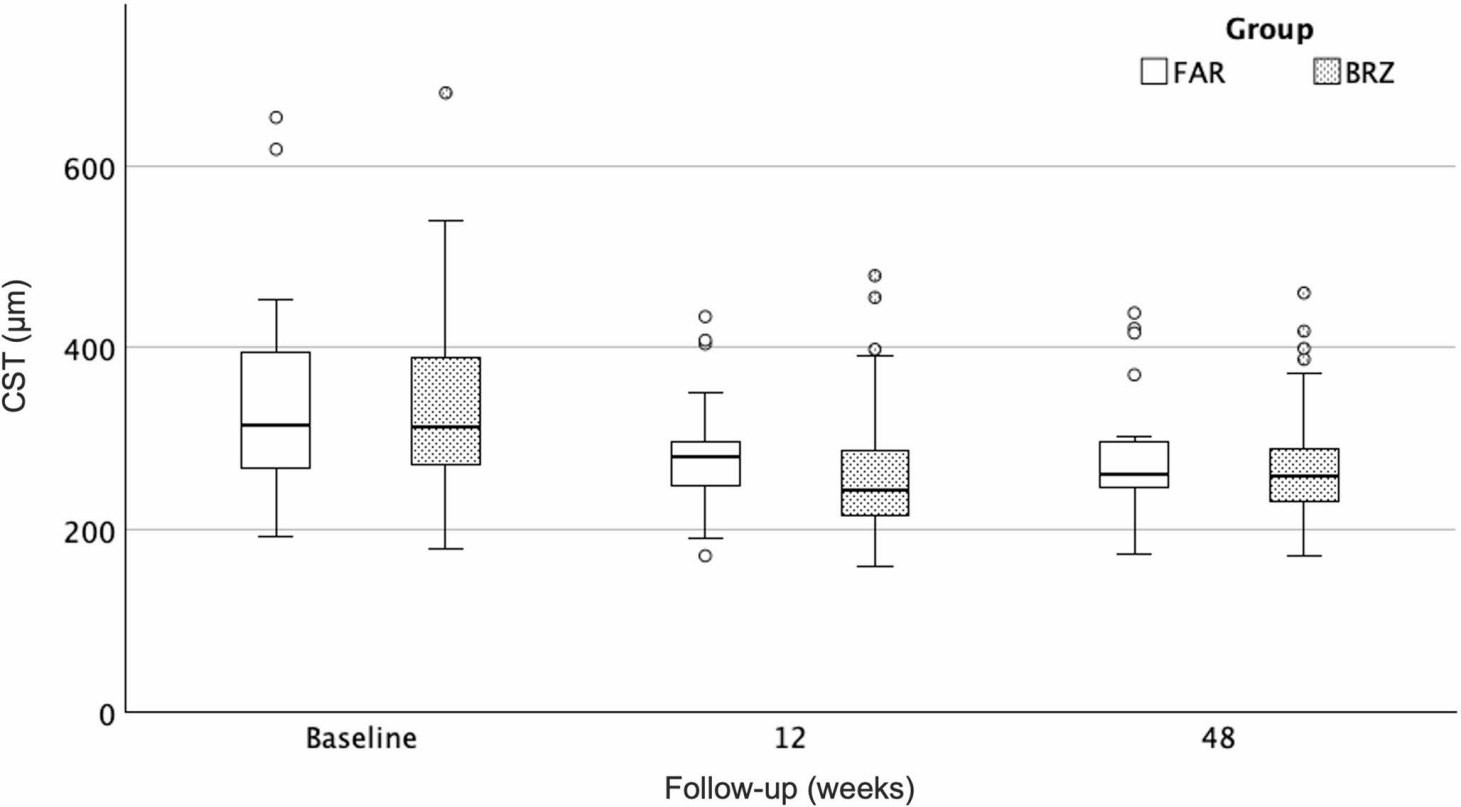
Boxplots showing central subfield thickness (CST, µm) at baseline, week 12, and week 48 after switch to BRZ or FAR. Data represent median ± IQR. Both agents achieved significant early CST reduction, which was sustained through 48 weeks, with no significant inter-group difference

**Fig. 3:**
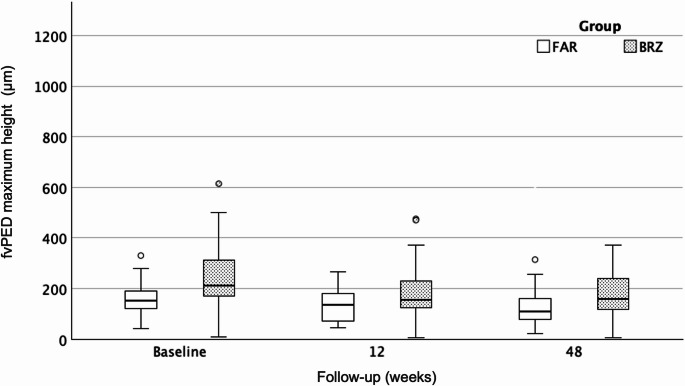
Boxplots showing maximum height of fibrovascular pigment epithelial detachment (fvPED, µm) at baseline, week 12, and week 48 after switch to BRZ or FAR. Data represent median ± IQR. FAR achieved a significantly greater reduction in fvPED height at week 48 compared with BRZ (*p* = 0.022)

Both agents maintained stable CST reduction (259 μm [220–290] vs. 260 μm [245–298]; *p* = 0.50) (Fig. 2; Table 3). FAR achieved a significantly greater reduction in fvPED height (110 μm [IQR: 74–161] vs. 160 μm [IQR: 117–250]; *p* = 0.022) (Fig. 3; Table 3) and exhibited a numerically lower SRF rate (24% vs. 41.5%; *p* = 0.08), while IRF and SHRM rates were similar (*p* > 0.05) (Table 3). Although BRZ showed stronger short-term PED responsiveness, FAR demonstrated a significantly greater absolute fvPED reduction at week 48, with percentage changes indicating only a nonsignificant numerical advantage (Table 3).

The total number of injections during 48 weeks was significantly lower with BRZ (6.3 ± 0.8 injections vs. 7.2 ± 1.5 injections; *p* = 0.009), whereas the mean last achieved treatment interval was comparable (9.4 ± 1.7 weeks vs. 9.9 ± 3.5 weeks; *p* = 0.80). These findings are consistent with prior real-world reports showing reduced injection frequency after switching to BRZ [5, 6].

Two BRZ-treated eyes (4.9%) discontinued treatment due to mild IOI, which resolved with topical corticosteroids without vision loss. No IOI or vascular occlusive events occurred in the FAR group. The observed inflammation rate aligns with published real-world data indicating a low single-digit IOI incidence following BRZ administration [8].

## Discussion

In this real-world cohort of poor-responsive nAMD, switching to BRZ or FAR under a TAE regimen without an initial loading phase resulted in stable visual acuity, sustained anatomic improvement, and a meaningful reduction in treatment burden over one year. The mean injection interval was extended from approximately 5 weeks prior to switch to 9–10 weeks at week 48. Both agents achieved durable disease control with comparable mean treatment intervals. Short-term outcomes showed significant fluid reduction and stable BCVA in both groups, with BRZ providing a significantly greater early CST reduction and a significantly stronger percentage fvPED decrease, whereas FAR showed lower absolute fvPED height at week 12. Long-term outcomes demonstrated that CST remained stably reduced and comparable between agents, while FAR achieved a significantly greater absolute fvPED reduction at week 48, with percentage changes showing only a nonsignificant numerical advantage. BRZ maintained disease stability with significantly fewer injections but was associated with two mild cases of IOI, both resolving without vision loss.

These outcomes on BRZ align with real-world studies evaluating short-term switch outcomes. Hussain et al. [8] and Bulirsch et al. [21] reported early drying and CST improvement in poor-responsive nAMD. The large German REALIZE study by Liegl et al. [6], together with a U.S. cohort by Coney et al. [5] and the U.S.-based Intelligent Research in Sight (IRIS^®^) Registry by MacCumber et al. [22], demonstrated approximately a 3-week interval extension at 12 months in treatment-experienced patients requiring frequent injections, consistent with the durability observed in HAWK/HARRIER in treatment-naive patients [14]. For FAR, pivotal and real-world data consistently show robust anatomy and PED regression: a prospective Chinese cohort reported significant PED reduction after three injections [3], and post-hoc analyses of TENAYA/LUCERNE indicated greater serous PED resolution with FAR than aflibercept 2 mg, supporting a dual VEGF-A/Ang-2 effect on vascular stabilization and RPE re-attachment [16]; large real-world cohorts by Grimaldi et al. [9], Sim et al. [12], Weber et al. [13]) corroborated sustained vision, fluid/PED reduction, and moderate treatment interval extension after switching to FAR while conducting an initial upload. Therefore, the more pronounced fvPED regression observed with FAR is likely influenced, at least in part, by its dual VEGF-A/Ang-2 inhibition. Although intraocular Ang-2 levels in nAMD are comparatively low [16, 18, 23], experimental data suggest that Ang-2 expression may increase with greater RPE-detachment height, potentially amplifying the effect of Ang-2 inhibition in such cases [24].

It extends previous findings by showing that comparable outcomes can be achieved without re-loading, confirming that omission of an upload phase is feasible without compromising efficacy. These results complement pivotal trial evidence and align with emerging data such as the Light Touch Trial by Sivaprasad et al., supporting a practical, resource-efficient treatment approach that maintains visual stability while reducing patient and clinic burden.

To our knowledge, the only previous head-to-head comparison of BRZ and FAR was the short-term study by Kin et al., which reported comparable anatomical efficacy three months after switching from aflibercept 2 mg with a trend towards greater visual improvements and reductions in CST with BRZ [20]. Our study therefore represents the first long-term real-world comparison of both agents under a TAE regimen without an initial loading phase in poor-responsive nAMD. It extends previous findings by showing that comparable outcomes can be achieved without re-loading, confirming that omission of an upload phase is feasible without compromising efficacy. These results complement pivotal trial evidence and align with emerging data such as the ongoing Light Touch Trial by Sivaprasad et al. (initiated in June 2024; results pending), supporting a practical, resource-efficient treatment approach that maintains visual stability while reducing patient and clinic burden.

Both agents achieved comparable long-term anatomical control but showed different temporal profiles: BRZ provided a faster early CST reduction and maintained disease stability with fewer injections, whereas FAR was associated with more pronounced PED regression and a tendency toward better functional outcomes. Clinically, both agents were effective in controlling disease activity in poor-responsive nAMD. FAR may be preferred in eyes with large or persistent PEDs or in patients with prior IOI due to its favorable safety profile and PED effect [3, 12, 13, 16]. BRZ remains an effective option when reducing injection frequency is prioritized, provided that inflammation monitoring is ensured [5, 6, 22].

Limitations include the retrospective, single-center design with potential selection and observer bias and unequal group sizes (41 BRZ vs. 25 FAR). Baseline BCVA was slightly better in FAR (0.49 vs. 0.54 logMAR), possibly contributing to the minor functional difference at week 48, although OCT characteristics and pretreatments were comparable. The comparatively smaller FAR cohort should also be acknowledged as a potential source of under-powered functional comparison between groups. Follow-up was limited to one year, and real-world TAE decisions introduce variability compared with standardized trials. Adverse event reporting focused on clinically manifest IOI; subclinical inflammation cannot be excluded. Despite these limitations, internal consistency was high as both cohorts were managed identically and followed the same upload-free TAE protocol. Real-world long-term switch data for BRZ or FAR remain limited, particularly for upload-free strategies; our results help address this gap and are consistent with pivotal and real-world evidence [5, 6, 8, 9, 12, 14–16, 19–21, 25].

In conclusion, our study provides the first one-year real-world head-to-head comparison of BRZ and FAR in poor-responsive nAMD under a TAE regimen without an initial loading phase. Both agents maintained stable vision and anatomy at one year. FAR achieved significant PED reduction and showed a slight better functional trend. However, this should be interpreted with caution given the higher baseline BCVA in this group. In contrast, BRZ was associated with fewer injections while maintaining comparable treatment intervals. These findings support both agents as viable options for individualized long-term management of poor-responsive nAMD within a resource-efficient, upload-free TAE framework.

## Data Availability

All data supporting the findings of this study are included within the article. Detailed patient data, which are sensitive in nature, are not publicly available due to privacy and ethical restrictions but can be provided in anonymized form by the corresponding author upon reasonable request.

## Abbreviations

Ang-2: Angiopoietin-2
BCVA: Best-corrected visual acuity
CST: Central subfield thickness
fvPED: Fibrovascular pigment epithelial detachment
IRF: Intraretinal fluid
IOI: Intraocular inflammation
IOP: Intraocular pressure
IQR: Interquartile range
IRIS®: Intelligent Research in Sight
IVT: Intravitreal anti-VEGF therapy
SD-OCT: Optical coherence tomography
TAE: Treat and extend treatment
SD: Standard deviation
SD-OCT: Spectral-domain-optical coherence tomography
SRF: Subretinal fluid
VEGF: Vascular endothelial growth factor

## References

[1] Wong WL, Su X, Li X, Cheung CM, Klein R, Cheng CY, Wong TY (2014) Global prevalence of age-related macular degeneration and disease burden projection for 2020 and 2040: a systematic review and meta-analysis. Lancet Glob Health 2:e106–116. 10.1016/S2214-109X(13)70145-1 25104651 10.1016/S2214-109X(13)70145-1

[2] Holz FG, Tadayoni R, Beatty S, Berger A, Cereda MG, Hykin P, Staurenghi G, Wittrup-Jensen K, Altemark A, Nilsson J, Kim K, Sivaprasad S (2016) Key drivers of visual acuity gains in neovascular age-related macular degeneration in real life: findings from the AURA study. Br J Ophthalmol 100:1623–1628. 10.1136/bjophthalmol-2015-308166 27030279 10.1136/bjophthalmol-2015-308166 PMC5256408

[3] Yufeng X, Ningxi H, Mingzhi S, Weixin Z, Panpan Y (2025) Real-world outcomes of a loading phase with intravitreal faricimab in refractory neovascular Age-Related macular degeneration (nAMD) patients. BMC Ophthalmol 25:347. 10.1186/s12886-025-04212-7 40598116 10.1186/s12886-025-04212-7 PMC12211914

[4] Amoaku WM, Chakravarthy U, Gale R, Gavin M, Ghanchi F, Gibson J, Harding S, Johnston RL, Kelly SP, Lotery A, Mahmood S, Menon G, Sivaprasad S, Talks J, Tufail A, Yang Y (2015) Defining response to anti-VEGF therapies in neovascular AMD. Eye Lond 29:1397–1398. 10.1038/eye.2015.159 26446737 10.1038/eye.2015.159 PMC5176328

[5] Coney JM, Zubricky R, Sinha SB, Sonbolian N, Zhou L, Hull TP, Lewis SA, Miller DG, Novak MA, Pendergast SD, Pham H, Platt SM, Rao LJ, Schartman JP, Singerman LJ, Donkor R, Fink M, McCoy J, Karcher H (2023) Switching to brolucizumab: injection intervals and visual, anatomical and safety outcomes at 12 and 18 months in real-world eyes with neovascular age-related macular degeneration. Int J Retina Vitreous 9:8. 10.1186/s40942-023-00445-0 36726178 10.1186/s40942-023-00445-0 PMC9891747

[6] Liegl RG, Karcher H, Chetty-Mhlanga S, Igwe F, Freitas R (2023) The treatment patterns with brolucizumab in Germany (REALIZE) study: a retrospective cohort study based on longitudinal prescription data. Ophthalmol Ther 12:195–208. 10.1007/s40123-022-00596-7 36327000 10.1007/s40123-022-00596-7 PMC9834462

[7] Schneider M, Bjerager J, Hodzic-Hadzibegovic D, Klefter ON, Subhi Y, Hajari J (2024) Short-term outcomes of treatment switch to faricimab in patients with aflibercept-resistant neovascular age-related macular degeneration. Graefes Arch Clin Exp Ophthalmol 262:2153–2162. 10.1007/s00417-024-06421-0 38416237 10.1007/s00417-024-06421-0 PMC11222265

[8] Hussain RM, Neal A, Yannuzzi NA, Patel KH, Huo S, Hariprasad SM, Bhatia SP (2021) Brolucizumab for persistent macular fluid in neovascular age-related macular degeneration after prior anti-VEGF treatments. Ther Adv Ophthalmol 13:25158414211055964. 10.1177/25158414211055964 34926990 10.1177/25158414211055964 PMC8679013

[9] Grimaldi G, Ambresin A, Pfister IB, Schild C, Plasencia C, Hatz K, Stillenmunkes R, Munk MR, Paris A, Menghini M, Artemiev D, Ebneter A, Cattaneo J, de Oliveira Figueiredo EC, Eandi CM, Frohlich J, Feltgen N, Spitznagel T, Somfai GM, Cozzi M, Zweifel S, Weinberger A, Garweg JG (2025) One-year outcomes after switching to faricimab in eyes with pre-treated neovascular age-related macular degeneration: a Swiss retina research network report. Ophthalmol Retina DOI. 10.1016/j.oret.2025.03.015 10.1016/j.oret.2025.03.015 40139459

[10] Janmohamed IK, Mushtaq A, Kabbani J, Harrow S, Nadarajasundaram A, Ata A, Monye H, Jarrar Z, Hannan S, Membrey L (2025) 1-year real-world outcomes of faricimab in previously treated neovascular age-related macular degeneration. Eye Lond. 10.1038/s41433-025-03616-5 39863706 10.1038/s41433-025-03616-5 PMC12044001

[11] Jones N, Gore C, Saedon H, O’Donnell C, Mahmood S (2025) Efficacy of treatment with faricimab for patients with refractory nAMD. Eur J Ophthalmol 11206721251328097. 10.1177/11206721251328097 10.1177/11206721251328097 40134284

[12] Sim SY, Chalkiadaki E, Koutsocheras G, Nicholson L, Sivaprasad S, Patel PJ, Selvam S, Pal B, Keane PA, Bhatia B, Hamilton R, Moorfields Medical Retina Injection S (2025) Real-world 1-year outcomes of treatment-intensive neovascular age-related macular degeneration switched to faricimab. Ophthalmol Retina 9:22–30. 10.1016/j.oret.2024.07.020 39084554 10.1016/j.oret.2024.07.020

[13] Weber C, Schipper P, Stasik I, Weinhold L, Bulirsch L, Thiele S, Holz F, Liegl R (2024) Early real-world experience with intravitreal Faricimab for neovascular AMD: FAN study. AJO International 1:100074. 10.1016/j.ajoint.2024.100074

[14] Dugel PU, Singh RP, Koh A, Ogura Y, Weissgerber G, Gedif K, Jaffe GJ, Tadayoni R, Schmidt-Erfurth U, Holz FG (2021) HAWK and HARRIER: ninety-six-week outcomes from the phase 3 trials of brolucizumab for neovascular age-related macular degeneration. Ophthalmology 128:89–99. 10.1016/j.ophtha.2020.06.028 32574761 10.1016/j.ophtha.2020.06.028

[15] Heier JS, Khanani AM, Quezada Ruiz C, Basu K, Ferrone PJ, Brittain C, Figueroa MS, Lin H, Holz FG, Patel V, Lai TYY, Silverman D, Regillo C, Swaminathan B, Viola F, Cheung CMG, Wong TY, Investigators L (2022) Efficacy, durability, and safety of intravitreal faricimab up to every 16 weeks for neovascular age-related macular degeneration (TENAYA and LUCERNE): two randomised, double-masked, phase 3, non-inferiority trials. Lancet 399:729–740. 10.1016/S0140-6736(22)00010-1 35085502

[16] Chaudhary V, Mar F, Amador MJ, Chang A, Gibson K, Joussen AM, Kim JE, Lee J, Margaron P, Saffar I, Wong D, Wykoff C, Sadda S (2025) Emerging clinical evidence of a dual role for Ang-2 and VEGF-A blockade with faricimab in retinal diseases. Graefes Arch Clin Exp Ophthalmol 263:1239–1247. 10.1007/s00417-024-06695-4 39708087 10.1007/s00417-024-06695-4 PMC12148975

[17] Diack C, Avery RL, Cheung CMG, Csaky KG, Gibiansky L, Jaminion F, Gibiansky E, Sickert D, Stoilov I, Cosson V, Bogman K (2024) Ocular pharmacodynamics of intravitreal faricimab in patients with neovascular age-related macular degeneration or diabetic macular edema. Transl Vis Sci Technol 13:13. 10.1167/tvst.13.11.13 39535745 10.1167/tvst.13.11.13 PMC11562974

[18] Joussen AM, Ricci F, Paris LP, Korn C, Quezada-Ruiz C, Zarbin M (2021) Angiopoietin/Tie2 signalling and its role in retinal and choroidal vascular diseases: a review of preclinical data. Eye (Lond) 35:1305–1316. 10.1038/s41433-020-01377-x 33564135 10.1038/s41433-020-01377-x PMC8182896

[19] Lee JH, Shin JY, Ahn J (2025) First-year real-world experience of intravitreal Brolucizumab injection for refractory neovascular age-related macular degeneration. Jpn J Ophthalmol 69:43–48. 10.1007/s10384-024-01134-7 39496987 10.1007/s10384-024-01134-7 PMC11821690

[20] Kin A, Mizukami T, Ueno S, Mishima S, Shimomura Y (2024) Short-term comparison of switching to brolucizumab or faricimab from aflibercept in neovascular AMD patients. Medicina (Kaunas). 10.3390/medicina60071170 39064598 10.3390/medicina60071170 PMC11278758

[21] Bulirsch LM, Sassmannshausen M, Nadal J, Liegl R, Thiele S, Holz FG (2022) Short-term real-world outcomes following intravitreal Brolucizumab for neovascular AMD: SHIFT study. Br J Ophthalmol 106:1288–1294. 10.1136/bjophthalmol-2020-318672 33846161 10.1136/bjophthalmol-2020-318672 PMC9411904

[22] MacCumber MW, Wykoff CC, Karcher H, Adiguzel E, Sinha SB, Vishwakarma S, LaPrise A, Igwe F, Freitas R, Ip MS, Zarbin MA (2023) Factors linked to injection interval extension in eyes with wet Age-Related macular degeneration switched to Brolucizumab. Ophthalmology 130:795–803. 10.1016/j.ophtha.2023.03.017 36990322 10.1016/j.ophtha.2023.03.017

[23] Regula JT, von Lundh Leithner P, Foxton R, Barathi VA, Cheung CM, Bo Tun SB, Wey YS, Iwata D, Dostalek M, Moelleken J, Stubenrauch KG, Nogoceke E, Widmer G, Strassburger P, Koss MJ, Klein C, Shima DT, Hartmann G (2016) Targeting key angiogenic pathways with a bispecific CrossMAb optimized for neovascular eye diseases. EMBO Mol Med 8:1265–1288. 10.15252/emmm.201505889 27742718 10.15252/emmm.201505889 PMC5090659

[24] Paterson C, Cannon J, Vargis E (2023) The impact of early RPE cell junction loss on VEGF, Ang-2, and TIMP secretion in vitro. Mol Vis 29:87–101 37859808 PMC10584031

[25] Maehara Y, Notomi S, Shiose S, Fukuda Y, Kiyohara K, Kano K, Ishikawa K, Hisatomi T, Sonoda KH (2025) Switching to faricimab alleviates persistent subretinal fluid and pigment epithelial detachment in neovascular age-related macular degeneration. Jpn J Ophthalmol. 10.1007/s10384-025-01264-6 40844677 10.1007/s10384-025-01264-6

